# Examine medicinal plants from South Africa for suppression of *Meloidogyne incognita* under glasshouse conditions

**DOI:** 10.21307/jofnem-2020-029

**Published:** 2020-04-09

**Authors:** Mbokota Candy Khosa, Zakheleni Dube, Dirk De Waele, Mieke Stefanie Daneel

**Affiliations:** 1 Agricultural Research Council − Tropical and Subtropical Crops, Private Bag X11208, Mbombela 1200, South Africa; 2 School of Biology and Environmental Sciences, University of Mpumalanga, Private Bag X11283, Mbombela 1200, South Africa; 3 Unit for Environmental Sciences and Management, North-West University, Private Bag X6001, Potchefstroom 2520, South Africa; 4 Laboratory of Tropical Crop Improvement, Department of Biosystems, Faculty of Bioscience Engineering, University of Leuven (KU Leuven), Willem de Croylaan 42, 3001 Heverlee, Belgium

**Keywords:** Phytonematicide, plant growth, reproductive potential, root-knot nematodes, soil amendments

## Abstract

The nematicidal activity of crudely milled powders of stems, leaves, and bulbs of *Cassia abbreviata*, *Cissus cactiformis*, *Euphorbia ingens*, *Ipomoea kituiensis, Synadenium cupulare*, *Senna petersiana*, *Urigenia sanguinea*, *Maerua angolensis*, and *Tabernaemontana elegans* on eggs and J2 population densities of *Meloidogyne incognita* race 2 on tomato was examined under glasshouse conditions. These plant species have medicinal properties and are being used in South Africa by traditional healers as so-called “muti.” All plant species showed a suppressive effect. Relative to untreated control, the soil amendments consistently suppressed *M. incognita* population densities in tomato roots and the reproductive potential (RP) of the nematode. When compared to fenamiphos, a commercial systemic chemical nematicide, the soil amendments performed comparable or better in suppressing nematode populations in the root systems in 2008 and 2009, but fenamiphos performed better than all soil amendments in 2011. The RP of *M. incognita* was comparable for both soil amendment- and fenamiphos-treated plants. No consistent trend in the effect of the soil amendments on plant root and shoot bioweight was observed, except when plants were treated with *T. elegans*-based soil amendments and both root and shoot bioweight were consistently higher compared with untreated control plants. Our results show that the plant species examined are potential sources of phytonematicides effective against *M. incognita* race 2.

In South Africa (SA), the majority of the rural and peri-urban populations depend for their daily dietary food needs on vegetables, grains, and leguminous crops produced mainly in household and community gardens, and on smallholder farms (Aliber, 2009). In this resource-poor agricultural sector, available land is often limited and, therefore, frequently re-used, which not only degrades the quality of the soil but also aggravates soil disease and pest problems (Aliber, 2009^). Plant-parasitic nematodes, especially root-knot nematodes (*Meloidogyne* spp.), are worldwide an extremely important limiting factor in vegetable production (^Hallmann and Meressa, 2018).

Tomato (*Solanum lycopersicon* L.) is one of the most common vegetables grown in SA (FAO, 2017). Tomato can be infected by a wide variety of plant-parasitic nematodes but predominantly by root-knot nematodes which can cause significant yield losses (Jones et al., 2017). A nematode survey in rural and peri-urban households, community and school gardens, as well as smallholder fields in SA showed that root-knot nematodes are the predominant biotic factor affecting the production of vegetables: 48 of 51 sites sampled were infested with root-knot nematodes (Mtshali et al., 2002). Most of the commercial tomato varieties grown in SA are susceptible to *Meloidogyne incognita* race 2 and *M. javanica* (Fourie et al., 2001).

Nematicide application is usually effective in reducing plant-parasitic nematode population densities below damage threshold levels (Desaeger et al., 2017) in the short term but resource-poor peri-urban and rural tomato growers do not have the means to purchase nematicides which are often expensive. Also, many of these chemicals are banned from the market because they are highly toxic to animals and humans, posing a serious threat to the environment and dangerous to handle (Jones, 2017). To alleviate the nematode problem and secure food security, alternative low-input, accessible, affordable and environment-friendly nematode management practices need to be developed and implemented.

The need for discovering less toxic and environmental-friendly substitutes for commercial nematicides created an opportunity for alternative products such as phytonematicides (botanical nematicidals, bionematicides) (Ntalli and Caboni, 2012; Caboni and Ntalli, 2014). The basic materials for the development of phytonematicides were usually collected from locally available plants (Mashela et al., 2017). Fresh or dried, crudely milled, ground or infused plant material of, for instance castor bean (*Ricinus communis* L.) and wild cucumber (*Cucumis myriocarpus* Naudin) produce phytonematicides that can suppress root-knot nematode population densities (Mashela, 2002; Mashela and Nthangeni, 2002; Dandurand et al., 2017).

So-called “muti” are African traditional medicines that make use of various natural products derived from trees and other plant species. These botanical medicines are usually prescribed by an inyanga or herbal healer and they are mostly endemic to Southern Africa. In SA, traditional healers use these mutis at controlled dosage rates to treat humans and domestic animals for various ailments (Van Wyk et al., 2009). The medicinal effects of these muti lead us to the hypothesis that they might be toxic to small soil-dwelling organisms such as plant-parasitic nematodes. These muti, as well as supplies of dried and finely ground material made from them, are abundant in the rural areas of the lowveld in Mpumalanga, Limpopo, and KwaZulu-Natal provinces of SA. This discovery could be a significant contribution toward sustainable crop production in the resource-poor agricultural sector of these provinces. The objective of this study was to evaluate crudely milled powders of different parts of *Cassia abbreviata* Oliv. (sjambok pod), *Cissus cactiformis* Gilg. (cucumber cactus), *Euphorbia* *ingens* E. Mey. ex Boiss. (gewone melkboom, naboom), *Ipomoea kituiensis* (Burm.) Merr. (kitui morning glory), *Maerua angolensis* DC. (bead-bean tree), *Senna petersiana* (Bolle) Lock. (wild senna, monkey pod), *Synadenium cupulare* (Boiss.) Wheeler ex A. C. White (dead-man’s tree), *Tabernaemontana elegans* Stapf (toad tree), and *Urginea sanguinea* Shinz. (rooislangkop) on growth of tomato plants and suppression of population densities of *M. incognita* race 2 under glasshouse conditions.

## Materials and methodsCollection of plant material and preparation of powdered leaf meals

Stems, leaves, and bulbs of *C. abbreviata*, *C. cactiformis*, *E. ingens*, *I. kituiensis*, *M. angolensis*, *S. petersiana*, *S. cupulare*, *T. elegans*, and *U. sanguinea* were collected from traditional healers in the Mopani and Vhembe districts, Limpopo Province, SA. The botanical origin of the plant parts was identified and confirmed by a botanist from the South African National Biodiversity Institute (SANBI, Pretoria). The plant parts were chopped into 5-cm-long pieces and oven-dried for four days at 52°C prior to grinding in a Wiley mill and passed through a 1-mm-aperture sieve (Makkar, 2003). Crudely milled plant materials of 25 kg were stored in bulk in marked, air-tight glass containers at room temperature in the dark until they were used for the trials. *Cucumis myriocarpus* material, obtained from the Nematology Laboratory, University of Limpopo, Sovenga, SA, was included in the trials as a reference treatment (Mashela, 2002) together with the commercial nematicide fenamiphos. Soil amendments of *C. myriocarpus* had been reported to suppress the population densities of *M. incognita* (Mashela, 2002).

### Preparation of nematode inoculum

A population of *M. incognita* race 2, of which the identification was confirmed by sequence-characterized amplified regions-polymerase chain reaction (Zijlstra et al., 2000; Fourie et al., 2001), was obtained from the Agricultural Research Council (ARC)-Grain Crops research institute, Potchefstroom, SA, and multiplied during two months in a separate glasshouse on plants of the susceptible tomato cultivar Floradade. To obtain the inoculum, nematode eggs and infective second-stage juveniles (J2) were extracted from the tomato roots by shaking the roots in a 1% NaOCl solution followed by sieving (Hussey and Barker, 1973) and passage through a set of nested sieves with apertures from top to bottom of 150, 63, 38, and 25 μm.

### Trial set-up

The trials were carried out in a glasshouse at the ARC-Tropical and Subtropical Crops research institute in Mbombela, SA (25°27´06.18˝S, 30°58´05.21˝E). In 2006 and 2007, crudely milled powder of seven plant species (*C. abbreviata*, *C. cactiformis*, *E. ingens*, *I. kituiensis*, *S. petersiana*, *S. cupulare*, and *U. sanguinea*) were evaluated. Based on the first trial (2006 and 2007), the soil amendments of *C. abbreviata*, *I. kituiensis*, *S. petersiana*, and *U. sanguinea* showed phytotoxicity. The three most effective plant species (*C. cactiformis*, *E. ingens*, and *S. cupulare*) were selected for further study in 2008, 2009, and 2011, along with two newly selected plant species (*M. angolensis* and *T. elegans*). Two six-weeks-old seedlings of tomato cultivar Floradade were transplanted into 5-L 150-mm-diameter plastic bags filled with a steam-pasteurized sandy loam soil (84% sand, 14% silt, and 2% clay), and pH 5.8. Immediately after transplanting, all seedlings were inoculated with 3,000±20 eggs and J2 in 5-cm-deep holes around the seedling stem using a 20-mL plastic syringe. In all trials, soil treatments consisted of a soil amendment of only one concentration (5 g/pot) that translates to 0.1% in w/w (0.71 metric tons/ha) of crudely milled powder. In 2006 and 2007, only untreated plants were included as controls; in 2008 and 2009, untreated plants plus fenamiphos-treated plants (Nemacur15 GR^®^ supplied by Bayer SA, Johannesburg, SA; 5 g of the product equivalent to 170 kg/ha was applied) were included as controls. In 2011, untreated- and fenamiphos-treated plants, and a soil amendment of 5 g dried, milled leaf meal of *C. myriocarpus* Naudin were included as positive controls. The addition of *C. myriocarpus* enable us to compare the effects of the powders used in our trials with a powder having known nematicidal activity (Pelinganga, 2013). Treatments were replicated six times in each trial and arranged in a randomized complete block design. Plants were irrigated with 300 mL tap water every other day. All plants were sprayed with Malasol^®^/Malathion^®^ (mercaptothion 500 g/L) and Redspidercide^®^ (tetradifon 81 g/L) alternatively every two weeks to prevent aphid and red spider mite infestation, respectively.

### Data collection

The trials were terminated 65 days after inoculation of the seedlings. Stems were cut off at the soil surface. Stem height of the tallest of the two plants per pot was recorded and the fresh shoot weight was calculated as the average of the two plants per pot. The root systems were carefully removed from the soil, cleaned under running tap water, and the fresh root weight recorded. To extract the nematode eggs and J2, the root systems of the two plants were cut into 1-cm-pieces and mixed thoroughly. A sub-sample of 50 g was taken at random from the mixed roots and incubated for 4 min in 300 mL of a 1% NaOCl solution (Hussey and Baker, 1973). The nematode suspension was then topped up to 100 mL and stored in a cold room at 11±4°C until the eggs and J2 were counted using a compound microscope (10 × and 40 × magnification).

### Statistical analysis

The data from each trial were subjected to analysis of variance (ANOVA) using SAS/STAT statistical software (SAS Institute, 2008). The standardized residuals of each variable were tested for deviations from normality using Shapiro−Wilk’s test. Nematode numbers were log_10_(*x* + 1) transformed to remove the inherent variability prior to analysis. Treatment means were separated using Fisher’s protected least significant difference (LSD) calculated at *P* ≤ 0.05 (Snedecor and Cochran, 1980).

## Results

### Effect of soil amendments on nematode population densities

In 2006 and 2007, soil amendments of all seven plant species included in the trials significantly (*P* ≤ 0.05) suppressed the nematode population densities by 68 to 83% compared to untreated control (Table 1). For *C. cactiformis*, *E. ingens* and *S. cupulare* these suppressive effects were confirmed in 2008 and 2009. In 2008 and 2009, soil amendments of *M. angolensis* and *T. elegans* suppressed the nematode population densities significantly (*P* ≤ 0.05) by 87 to 88% compared to untreated control plants while a significant difference with fenamiphos was observed in 2008. The suppressive effect of *C. cactiformis*, *E. ingens*, *S. cupulare*, and *T. elegans* were confirmed in 2011 when soil amendments based on these plant species significantly (*P* ≤ 0.05) suppressed nematode population densities with 78 to 94% compared to untreated control and, with results of all of the above except for *M. angolensis* being comparable to results with *C. myriocarpus*. The reproductive potential (RP) of *M. incognita* were comparable in both fenamiphos and soil amendment treated plants (Table 2).

**Table 1.** Reproduction of *Meloidogyne incognita* race 2 on tomato cv. Floradade plants treated with soil amendments of nine plant species at 65 days after inoculation with 3,000 eggs and second-stage juveniles (J2).

	Eggs + J2/root system				
Plant species	2006	2007	2008	2009	2011
*Cassia abbreviata*	3 925 b	2 050 d	−	−	−
*Cissus cactiformis*	2 500 cd	1 700 d	662 c	1 000 b	1 688 bc
*Euphorbia ingens*	2 500 cd	3 562 b	862 bc	788 cd	1 838 bc
*Ipomoea kituiensis*	2 575 cd	3 000 bc	−	−	−
*Synadenium cupulare*	2 125 d	2 538 cd	762 bc	562 d	2 588 bc
*Senna petersiana*	3 125 bc	2 088 d	−	−	−
*Urginea sanguinea*	2 775 cd	3 600 b	−	−	−
*Maerua angolensis*	−	−	650 c	688 cd	6 687 b
*Tabernaemontana elegans*	−	−	712 c	650 bcd	3 588 bc
*Cucumis myriocarpus*	−	−	−	−	2 550 c
Fenamiphos	−	−	1 050 b	875 bc	138 d
Untreated control	12 225 a	13 238 a	5 388 a	5 188 a	30 063 a

Notes: Column means followed by the same letter are not significantly (*P* ≤ 0.05) different according to Fisher’s least significant difference (LSD) test; means are average of six replications; gall index was not determined

**Table 2.** Reproductive potential (RP) of *Meloidogyne incognita* race 2 on tomato cv. “Floradade” plants treated with soil amendments at 65 days after inoculation.

Plant species	2006	2007	2008	2009	2011
*Cassia abbreviata*	1.3 b	0.7 bc	−	−	−
*Cissus cactiformis*	0.8 b	0.6 c	0.2 b	0.2 b	0.6 bc
*Euphorbia ingens*	0.8 b	1.2 b	0.3 b	0.2 b	0.6 bc
*Ipomoea kituiensis*	0.9 b	1.0 bc	−	−	−
*Synadenium cupulare*	0.7 b	0.8 bc	0.3 b	0.1 b	0.9 bc
*Senna petersiana*	1.0 b	0.7 bc	−	−	−
*Urginea sanguinea*	0.9 b	1.2 b	−	−	−
*Maerua angolensis*	−	−	0.2 b	0.2 b	2.2 b
*Tabernaemontana elegans*	−	−	0.2 b	0.1 b	1.2 bc
*Cucumis myriocarpus*	−	−	−	−	0.9 bc
Fenamiphos	−	−	0.4 b	0.2 b	0.0 bc
Untreated control	4.1 a	4.4 a	1.8 a	1.4 a	10.0 a
LSD	0.7165	0.6114	0.1415	0.1273	2.0788
*P*-value	≤ 0.001	≤ 0.001	≤ 0.001	≤ 0.001	≤ 0.001
*F*-value	19.97	34.84	137.72	108.15	15.20

Notes: Column means followed by the same letter are not significantly (*P* ≤ 0.05) different according to Fisher’s least significant difference (LSD) test; means are average of six replications; reproductive potential (RP) = Eggs  +  J2 in root system ÷ total fresh root weight (g)

### Effect of soil amendments on plant growth

There were no consistent trends in the effects of soil amendments on plant root and shoot weight. A significant (*P* ≤ 0.05) increase in fresh root weight compared to untreated control plants was observed in the trials carried out from 2007 until 2011 in which soil amendments of *C. cactiformis*, *E. ingens*, *S. cupulare*, *M. angolensis*, and *T. elegans* were included (Table 3). A significant (*P* ≤ 0.05) increase in fresh shoot weight compared to untreated control plants were observed in all the trials carried out from 2007 until 2011 for soil amendments of *T. elegans* and *C. cactiformis* (Table 3). Soil amendments of *E. ingens* and *S. cupulare* resulted in a significantly (*P* ≤ 0.05) higher fresh shoot weight compared to untreated control plants. *Marua angolensis* resulted in a significantly higher shoot weight in 2008 and 2011.

**Table 3.** Fresh root and shoot weight of tomato cv. Floradade plants treated with soil amendments of nine plant species at 65 days after inoculation with 3,000 eggs and second-stage juveniles (J2) of *Meloidogyne incognita* race 2.

	Fresh root weight (g)					Fresh shoot weight (g)				
Plant species	2006	2007	2008	2009	2011	2006	2007	2008	2009	2011
*Cassia abbreviata*	2.8 a	3.0 b	−	−	−	3.7 d	3.7 c	−	−	−
*Cissus cactiformis*	2.9 a	2.8 bc	30.2 a	20.6 a	14.1 d	9.2 a	8.6 a	9.9 ab	10.0 a	83.7 a
*Euphorbia ingens*	2.0 a	1.8 d	23.3 a	19.0 a	−	7.2 b	7.7 a	8.6 bc	8.1 abc	80.5 a
*Ipomoea kituiensis*	3.2 a	4.6 a	−	−	15.2 d	3.3 d	3.7 c	−	−	−
*Synadenium cupulare*	2.4 a	2.1 cd	22.1 bc	20.2 a	19.2 ab	5.3 c	6.4 b	7.6 c	9.2 ab	70.2 c
*Senna petersiana*	2.6 a	1.8 d	−	−	−	4.0 d	3.8 c	−	−	−
*Urginea sanguinea*	2.5 a	2.5 bcd	−	−	−	4.0 d	3.9 c	−	−	−
*Maerua angolensis*	−	−	25.3 b	14.9 b	17.6 bc	−	−	8.7 bc	7.1 bc	81.5 a
*Tabernaemontana elegans*	−	−	30.8 a	19.5 a	15.1 d	−	−	10.0 a	9.8 a	79.2 ab
*Cucumis myriocarpus*	−	−	−	−	20.0 a	−	−	−	−	68.1 cd
Fenamiphos	−	−	30.7 a	22.3 a	15.9 cd	−	−	10.0 a	10.4 a	73.3 bc
Untreated control	2.2 a	1.8 d	21.6 c	14.6 b	11.2 e	3.4 d	4.1 c	7.9 c	6.0 c	61.5 d

Notes: Column means followed by the same letter are not significantly (*P* ≤  0.05) different according to Fisher’s least significant difference (LSD) test; means are average of six replications

## Discussion

Based on the results of all five trials, this study showed that soil amendments of crudely milled powders of all plant species examined suppressed population densities of *M. incognita* race 2 in tomato roots. Unfortunately, gall indices were not determined which could have confirmed our results. Root gall rating should be included in further studies with soil amendments of crudely milled powders. The suppressive effect of the soil amendments of *C. cactiformis*, *E. ingens*, *S. cupulare*, *M. angolensis*, and *T. elegans* was mostly comparable to fenamiphos in 2008 and 2009 but not in 2011 when the reproduction of *M. incognita* was two- and three-fold higher in 2011 than 2009 and 2008, respectively. However, in 2011, the fresh and dry weights of tomato plants grown in soil treated with the soil amendments were higher compared with the untreated control plants, which may have resulted in higher nematode population densities in the root systems of the treated plants. When the nematode RP (RP = Eggs  +  J2 in root system ÷ total fresh root weight (g)) was considered, the suppressive effect of all soil amendments was comparable to fenamiphos in 2008, 2009, and 2011. Some inconsistencies were observed but they were few and inconsistencies are not surprising in this type of trials (Mashela et al., 2017). Nonetheless, managing nematodes with these compounds when nematode population densities are still below the economic threshold level should be further evaluated under field conditions.

During this study, phytotoxicity of some of the soil amendments included in the trials on the plant growth variables examined was observed and therefore some plant species such as *C. abbreviata*, *I. kituiensis*, *S. petersiana*, and *U. sanguinea* were replaced with *T. elegans* and *M. angolensis*. It can also not be excluded that at higher concentrations, the crudely milled powders may have a phytotoxic effect and this aspect should be closely monitored in further studies aimed at the development and use of these plant materials as phytonematicides. By investigating higher dosages in the field, the potential for replacement of Class I nematicides as nematode control agents can be evaluated, especially since many of the crudely milled powders performed similar to fenamiphos at a concentration which is considerably lower than the 1% w/w amendment rate which is often practical in the field (Mashela, 2002). However, if lower dosages can be suppressive, this will put less pressure on producing the plant material as it is time consuming to grow medicinal plants in large quantities.

Although toxicity of medicinal plants to humans or animals are not usually of a concern (Mashela et al., 2017), this aspect should be monitored closely in future studies. It is known that *R*. *communis* which shows great nematicidal activity is also very poisonous to humans (Mashela et al., 2017).

The phytochemical compounds of phytonematicides can be broadly classified as alkaloids, alkamides, carbohydrates, cyanogenic glycosides, fatty acids, glucosinolates, non-protein amino acids, phenolic compounds (coumarins, flavonoids, phenylpropanoids, tannins), polyacetylenes, polyketides, terpenoids, thiophenes, and waxes (Ntalli and Caboni, 2012; Caboni and Ntalli, 2014; Mashela et al., 2017). However, the nature and the mode of action of the nematicidal substance(s) of *C. cactiformis*, *E. ingens*, *S. cupulare*, *M. angolensis*, and *T. elegans* remained unknown and required further investigation.

This study confirmed that more plant species contain nematicidal compounds against root-knot nematodes (*in casu M. incognita* race 2) than previously reported. Products of some of these medicinal plants apparently have the potential to increase tomato growth and yield in root-knot nematode infested soil. The development of these plant materials as phytonematicides merits further investigation under microplot and field conditions. These plant materials may offer an accessible and affordable management alternative for root-knot nematode problems in household and community gardens, and smallholder fields especially in Mpumalanga, Limpopo, and KwaZulu-Natal provinces of SA where some of these medicinal plants such as *C. cactiformis*, *M. angolensis*, and *T. elegans* are grown abundantly. Possible human, animal, and environmental risks of their use should be determined, preferably in consultation with the traditional healers that use these materials for medicinal purposes.
